# Endangered with High Dispersal Abilities: Conservation Genetics of *Himantoglossum metlesicsianum* (Teschner) *P. Delforge* (Orchidaceae) in the Canary Islands

**DOI:** 10.3390/plants14121862

**Published:** 2025-06-17

**Authors:** Rocío González Negrín, Victoria Eugenia Martín Osorio, Pedro A. Sosa, Priscila Rodríguez-Rodríguez

**Affiliations:** 1 Departamento de Botánica, Ecología y Fisiología Vegetal, Facultad de Farmacia, Universidad de La Laguna, 38200 San Cristóbal de La Laguna, Santa Cruz de Tenerife, Spain; vemartin@ull.edu.es; 2 Instituto Universitario de Estudios Ambientales y Recursos Naturales, Universidad de Las Palmas de Gran Canaria, Campus de Tafira, 35017 Las Palmas de Gran Canaria, Spain; pedro.sosa@ulpgc.es (P.A.S.); priscila.rodriguez@ulpgc.es (P.R.-R.)

**Keywords:** conservation genetics, endemic, microsatellite markers, seed dispersal

## Abstract

*Himantoglossum metlesicsianum* is a threatened orchid with low population numbers and fragmented distribution, present in four of the Canary Islands. This study focused on assessing the genetic variability and population genetic structure of the natural populations known to date, identifying those characteristics of the species that condition the flow and genetic variation. For that purpose, we collected samples from eight sites in its distribution range and developed 14 polymorphic microsatellite markers. Despite its rarity, this orchid presents high levels of genetic diversity and a homogeneous population structure, characterised by a low degree of genetic differentiation and patterns consistent with high genetic connectivity among populations. Our results suggest that the species might show dichotomy in seed dispersal, combining long- and short-distance events. In addition, it is possible that pollen cross-pollination (pollinia) between adjacent sites may also be involved. In conclusion, these findings reveal unexpectedly high genetic diversity and connectivity among populations, despite the species’ rarity and fragmented distribution, highlighting key biological traits that should be considered in future conservation and recovery plans.

## 1. Introduction

Oceanic islands are natural laboratories for studies of plant evolution [1,2,3]. A characteristic feature of oceanic island floras is the large number of endemic species found within small areas. Due to spatial isolation and temporal boundaries, oceanic archipelagos provide an ideal system for studying the evolutionary processes involved in population differentiation and speciation [4,5,6,7]. The Canary Islands archipelago stands out for its rich biodiversity with a high rate of endemicity, belonging to the Mediterranean Biodiversity hotspot [8], and located in the Macaronesian Biogeographic region, off the northwestern coast of Africa.

The geographical origins of the endemic plants of the Canary Islands are primarily Mediterranean (35%), Northeastern African (25%), Eastern and Southern African and from the New World (22%), while the remaining 18% are derived from Macaronesia (including the Canaries), highlighting the important role of intra-archipelago diversification [9]. These patterns reflect a complex colonisation history and highlight the archipelago’s role in promoting evolutionary diversification. Recent evolutionary frameworks suggest that the high levels of endemism and genetic diversity observed in Canarian plants are not solely the result of allopatric speciation and long-term isolation, but also of more dynamic processes such as recurrent hybridisation and the formation of syngameons—groups of closely related species capable of interbreeding—which have been promoted by the geological ontogeny of the islands [10]. Geological changes such as erosion, volcanic collapse, and island rejuvenation have repeatedly reshaped habitats, fostering secondary contact and genetic admixture among previously isolated lineages, thus enhancing diversification.

Within this evolutionary framework, *Himantoglossum metlesicsianum* (Teschner) P. Delforge stands out as a notable case, with a biogeographical origin linked to both the Mediterranean region and North Africa [11].

Island species often consist of small and isolated populations, characterised by restricted gene flow and increased incidence of inbreeding, which makes them strongly affected by genetic drift. As a result, low genetic diversity is expected in island populations, which has implications for their long-term survival potential [12,13]. A loss of genetic diversity is commonly associated with reduced fitness [14,15], and populations with low levels of genetic diversity are expected to be less capable of adapting to environmental changes [16], thus placing a strong emphasis on the conservation of endemic island species.

The characterisation and structure of genetic diversity are fundamental tools for the conservation of threatened species, particularly in one of the plant families with the highest number of threatened species: orchids. Currently, half of the 1641 taxa assessed in the IUCN Red List fall into the Vulnerable, Endangered, or Critically Endangered categories [17,18]. Among the total taxa of the *Orchidaceae* family assessed in the list, 55% are classified as endangered [19] due to direct habitat loss, plant smuggling, the widespread impact of global climate change [20], and overexploitation for horticultural purposes [21]. Many species have limited ecological preferences, making them vulnerable to habitat loss and climate change, resulting in population declines and changes in species distribution [22,23].

A characteristic feature of the *Orchidaceae* family is that these plants produce a large number of dust-like seeds [24]. Due to their small size, orchid seeds can be dispersed over long distances by wind. These long-distance dispersal events are crucial for maintaining species and preserving genetic diversity in fragmented landscapes [25,26,27,28]. While some authors suggest that orchid seeds can travel distances of up to 2000 km [29], others have shown that most dispersal events occur over short distances [30,31], with seeds typically dispersing over a limited range and falling close to the mother plants. This pattern aligns with a leptokurtic distribution [32]. Moreover, the high gene flow among orchid populations is attributed to the ability of seeds to traverse long distances to colonise new environments [33], resulting in low genetic differentiation between populations. Orchids have evolved by developing specialised pollination strategies, primarily due to their floral characteristics: pollen is packaged in sacs known as pollinia, which ensures efficient pollen transfer, even when visitation rates by pollinators are low [34]. Additionally, there are orchid species that exhibit deceptive pollination strategies; these orchids do not provide a reward (nectar) to their pollinators [35,36,37]. These species exhibit pollination systems that do not offer rewards to their pollinators, which influences the genetic diversity and structure of populations [38]. In fact, species of the genus *Himantoglossum* have inflorescences characterised by a deceptive food strategy; they do not produce nectar but attract a wide range of pollinators [39].

The genus *Himantoglossum* is distributed across the Euro-Asiatic region, Northern Africa, the United Kingdom, and the Canary Islands. Orchids belonging to this genus are commonly known as “lizard orchids”; these plants produce inflorescences with striking flowers, characterised by a large and complex labellum, which in most species is highly twisted and follows a remarkable floral ontogeny [40]. The genus comprises approximately nine accepted species. Phylogenetic studies [41] showed that the Canary Island endemic *H. metlesicsianum* is a sister species to and divergent from *Himantoglossum robertianum*, appearing within the *robertianum* group [42].

*Himantoglossum metlesicsianum* is a threatened endemic orchid with eight populations in the archipelago, located on Tenerife, La Palma, Gran Canaria and El Hierro. In general, these populations are found in the western parts of the islands. In Tenerife, there are five populations, three of which are located in the west while the remaining two are located to the east, in Güímar and Arafo [43,44,45,46].

The species is found in areas of pine forest and occurs in semi-abandoned or abandoned plots of almond trees and vineyards. Generally, the populations have been affected by wildfires [47]. The potential vegetation surrounding the populations corresponds to a pine forest of *Pinus canariensis* [48], characterised as a dry-mesomediterranean thermophytic environment, with annual precipitation between 450 and 600 mm and average annual temperatures of 10–15 °C, experiencing occasional frosts and snowfall in winter, located between 900 and 1400 m above sea level. It thrives in biotopes where light is filtered through the tree canopy or tall shrub cover. Occasionally, the pine forest is influenced by fog, contributing considerable atmospheric moisture [49].

The total census for the Canary Islands in 2020–2021 recorded 304 individuals, with less than 4% of them developing flowers [47]. The life cycle of the species lasts seven months, beginning in October and ending in April with fruit dehiscence. This species reproduces by seeds, which have a fusiform and oblong shape. The seeds are small (380–590 μm long) with oblique and transverse striations and branching patterns on their periclinal surfaces. Field studies reveal the presence of individuals in very close proximity to one another, which could be attributed to two main factors: first, the availability of mycorrhizae in the soil; and second, the possibility that, due to a short-distance dispersal mechanism, the individuals are genetically related and located near the mother plants [47] such as other species of orchids [50,51]. Pollination is entomophilous, mainly carried out by *Bombus canariensis*, while seed dispersal is anemochorous [49]. The fruit capsules are dehiscent and contain tiny seeds.

The main objectives of this work consist of the genetic characterisation of the endemic *Himantoglossum metlesicsianum* and the identification of genetic differences among its natural populations. The study aims to (1) analyse the genetic structure of the plant’s populations, (2) estimate their levels of genetic diversity, (3) interpret the dispersal patterns of the species, and (4) develop the necessary guidelines and strategies for a recovery plan to ensure the species’ survival.

## 2. Results

The fourteen primer pairs were polymorphic for populations and subpopulations (See Table S1 in the Supplementary Material). Null alleles were detected in the populations: Hm08 (HAR = 0.22; HGC = 0.11; HLP = 0.22), Hm04 (HBB = 0.16; HAR = 0.19), Hm30 (HAR = 0.28; HGC = 0.23), Hm38 (HSA = 0.214; HCH = 0.18) and Hm45 (HGU = 0.17). In the initial analysis, one locus was excluded due to the presence of null alleles across all populations. HGC, HLP and HAR showed deviation from Hardy–Weinberg equilibrium. Significance tests for linkage disequilibrium at all loci indicate no disequilibrium.

The highest values of genetic diversity calculated for *H. metlesicsianum* were found on Tenerife, specifically in HBB (H_e_ = 0.695). On the other hand, La Palma presented the lowest genetic diversity (H_e_ = 0.503). The average of rarefied allelic richness over all loci ranged from 3.37 (HLP) to 2.34 (HSA). At the island level, Tenerife has a higher number of private alleles (HAR: 2, HBB: 4, HCH: 4, HGU: 1 and HSA: 6). HGC has four, and HLP has only one private allele. The maximum value detected for selfing rates was 20% for the subpopulation of Santiago del Teide (Table 1).

The Analysis of Molecular Variance (AMOVA) at the island scale indicated that the populations studied are relatively homogeneous from a genetic point of view (Table 2). The analysis shows a differentiation between islands of 7.81%. In addition, significant differences were detected between subpopulations located in the west and east of Tenerife. However, the percentage of variation was low (2.76%), with greater differentiation between populations within groups (6.94%).

Pairwise *F_ST_* values ranged from 0.034 (between HBB or HCH and HSA) to 0.157 (between HGU and HLP) (See Table S2 in the Supplementary Material). Regarding the PCoA, the first two axes accounted for 17.29% of proportion of the total variance (17.29%), with 9% explained by the first axis and 8.29% by the second (See Figure S1 in the Supplementary Material).

**Figure 1:**
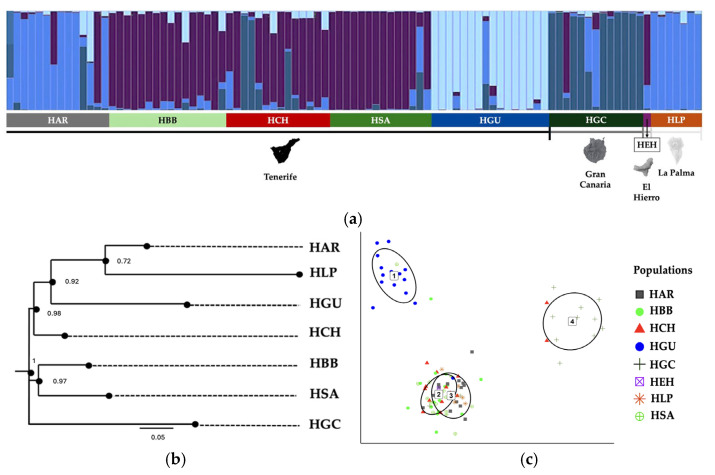
(**a**) Bar chart of the coancestry ratio inferred by Bayesian clustering analyses in STRUCTURE K = 4. (**b**) Neighbour-joining dendrogram from Nei’s genetic distance matrix; HEH is not included in this figure, as the corresponding sampling point was represented by a single individual. (**c**) Discriminant Principal Component Analysis (DAPC), the numbers 1 to 4 indicate the different DAPC groups.

This revealed the genetic relationship between the populations located to the west of Tenerife. The populations located to the east of Tenerife appear relatively distant from the rest. The most genetically distant populations are those located on La Palma and Gran Canaria. In the (DAPC) analysis, four clusters were estimated, showing an undefined population structure, with individuals being admixed in the four clusters (Figure 1c).

The neighbour-joining dendrogram represented HGC as closest genetically to the subpopulations located to the west of Tenerife (HSA and HBB). HGC has a more basal position in the tree (Figure 1b).

Bayesian structure analysis identified four genetic groups according to ΔK (K = 4) (See Table S3, Figures S2 and S3 in the Supplementary Material), as well as the DAPC analysis (Figure 1c). The western populations of Tenerife: Guía de Isora (HBB and HCH) together with HSA, show a high homogenisation of genes, this being one of the groups that appears clearly defined, which reflects a high degree of gene flow between these groups. Likewise, these subpopulations share genes with HGC, a population that appears as a defined group, differentiated from the rest. HG, which despite being located to the east of Tenerife, is defined as a group differentiated from the rest of the populations and subpopulations. HAR and HLP also appear in the same cluster (Figure 1a).

**Figure 2:**
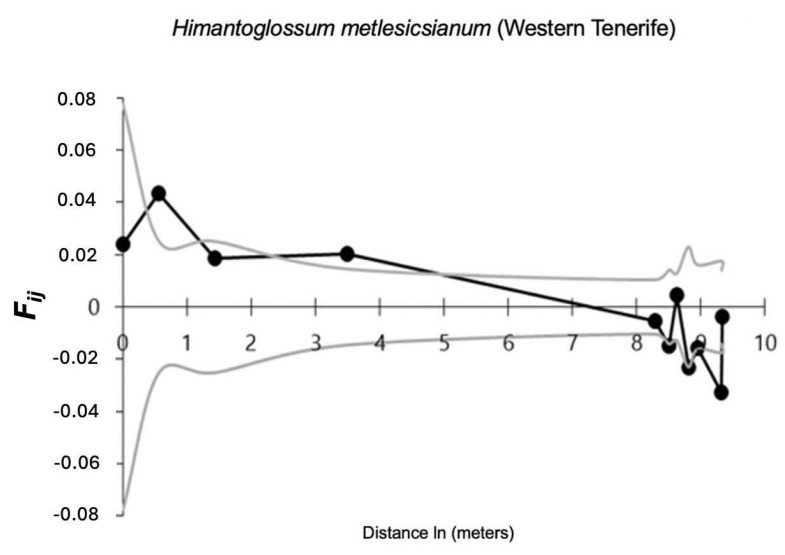
Coancestry coefficient according to Loiselle et al. (1995) [52]. Mean kinship coefficients (F_ij_) between pairs of individuals from each population as a function of linear geographic distance. The grey lines represent 95% confidence intervals, and the points outside these margins indicate a significant deviation from a random spatial distribution (*p* < 0.05). The black line shows observed values; grey lines indicate the 95% confidence envelope from random permutations. Values outside the envelope suggest significant spatial genetic structure.

In SGS analysis, F1 values were significant (*p* < 0.05), meaning that there is spatial autocorrelation in the first distance class at less than one meter (0.55 m). However, these values lie outside the confidence interval only at the second distance class. Although the result is significant, the value of the kinship index in the first distance class was low (Figure 2) [52].

## 3. Discussion

In this study, we have conducted the genetic characterisation and structural analysis of the species *Himantoglossum metlesicsianum.* Despite being a plant species with a restricted distribution, it does not exhibit a low level of genetic diversity, and homogenisation is detected among the studied populations.

Our analyses identified moderate to high levels of genetic diversity among the studied populations, which is noteworthy for a species considered endangered due to its limited populations and low number of individuals. The highest values of genetic diversity parameters were found in two sample sites on Tenerife (HBB and HSA), while the lowest value was detected in the population located on La Palma. This high genetic diversity in the islands may be influenced by the biotic characteristics of the lineage. These characteristics, along with the abiotic factors of each island, drive the diversification of the Canary Islands’ flora. The most suitable taxa for diversification in the Canary Islands’ abiotic landscape are those with high basic chromosome numbers, polyploidy, partial or total self-incompatibility, and long-distance seed dispersal [53], as is the case with the species under study, whose seeds can travel long distances.

On the other hand, it is expected that rare and endemic species, as well as those with restricted geographical distributions, exhibit lower levels of genetic diversity compared to widespread species [54]. However, this study may support the findings of Francisco-Ortega et al. (2000) [55] and De Paz and Caujapé-Castells (2013) [53] regarding the endemic species of the Canary archipelago, which are characterised by unexpectedly high genetic diversity. When comparing this species to other endemic Canary species, such as *Bencomia caudata* (H_e_ = 0.62), *Phoenix canariensis* (H_e_ = 0.60), or *Silene nocteolens* (H_e_ = 0.78) [56], it is evident that the values for *Himantoglossum metlesicsianum* fall within a similar range to the taxa studied in this review (H_e_ = 0.633).

In general, existing data on the continental genus *Himantoglossum* shows low genetic diversity; for instance, *H. hircinum* [23] exhibited a low degree of genetic diversity as populations moved away from their centre of distribution. Conversely, in a comparative study of various orchid populations from different species, *Himantoglossum affine* displayed the highest genetic diversity values [57]. Although these studies are not directly comparable to the species under investigation, as they were not conducted using codominant molecular markers, they provide insights into the genetic diversity patterns and behaviours of the species at a continental level.

It is remarkable that there are populations of *Himantoglossum metlesicsianum* that are in Hardy–Weinberg equilibrium despite the low population sizes. However, there are others that are in Hardy–Weinberg disequilibrium (HBB, HCH, HGC, and HSA). Generally, in the *Orchidaceae* family, these results are associated with the type of seed dispersal and morphology. Regardless of the long-distance dispersal capacity of orchids, seed dispersal may also exhibit a limited distribution range, close to the maternal individuals of a population, leading to inbreeding processes [58]. For other species of *Himantoglossum*, such as *H. hircinum*, it was estimated that the average distance seeds travel is only one meter [59]. Regarding offspring, these new seeds may recruit near the parent plants, resulting in a deficiency of heterozygous individuals.

One of the objectives of this study is to determine the degree of differentiation among populations of *Himantoglossum metlesicsianum.* Population genetic structure can be analysed using different indices, such as genetic differentiation or gene flow. The various analyses implemented, such as Bayesian analysis in STRUCTURE and DAPC for the entire sample set, detected four defined groups. Despite the identification of these four clusters, there is some mixing among them, as illustrated in the PCoA figure. These results are consistent after determining the hierarchical structure of the population with AMOVA, which estimated a low percentage of differentiation between islands (7.81%) and at the insular level for Tenerife (2.76%). The difference in differentiation percentages is low (>5%), indicating no notable insular effect on genetic structure. These results may conclude that long-distance seed dispersal is not hindered, allowing the species to easily find opportunities for successful dispersal and germination to colonise other islands.

Furthermore, the *F_ST_* values were statistically significant. These values were moderate for populations that are geographically distant, such as the population of La Palma compared to HGC (*F_ST_* = 0.157), where the geographical distance between them is 230 km, resulting in a decrease in specific gene flow between them. Additionally, at the insular level, the populations in eastern Tenerife (HGC and HAR) do not appear to be related to each other, despite being less than four kilometres apart, which may be influenced by physical barriers detected in the habitat. For instance, in the case of HGU, the individuals within it are found in a restricted distribution area, confined by the steep walls of a ravine [47]. Consequently, the seed dispersal capacity carried by the wind may be influenced by wind dynamics and local conditions, such as habitat structure and continuity. Another aspect that may affect seed dispersal is the wooded area present in this locality, characterised by the tree species *Pinus canariensis*. Some authors have already found negative effects on orchid dispersal related to the height of surrounding plants, as well as physical barriers caused by local topography [31].

Regarding HLP, it is related to the subpopulations located to the east of Tenerife, despite the greater distance between these populations compared to those to the west of Tenerife. HGU shows a closer relationship with the populations situated to the west of Tenerife. This may be related to occasional or rare long-distance seed dispersal events by wind [24], with a geographical distance of 104 km between these points. Although we cannot discard the existence of non-discovered populations or even extinct populations that connected the distribution in the past.

In general, a poorly differentiated population genetic structure is expected in the *Orchidaceae* family. Orchids produce small, very light seeds with an average size of 380–590 μm in a “dust” form, which can float in the air over long distances, facilitating gene exchange between populations [29]. Therefore, the lack of speciation processes may be explained by extensive gene flow among subpopulations, leading to a weak population structure throughout the archipelago [60].

The pollen crossing system is another variable that may be influencing the genetic structure of the species. The life history characteristics of species, particularly reproduction, have a significant effect on both genetic diversity and population structure [61,62,63]. Studies conducted by Cozzolino and Widmer (2005) [38] indicate that there are differences in genetic diversity and structure between orchid species that provide rewards and those that do not offer rewards to their pollinators. These authors have demonstrated that orchids with high genetic diversity within populations and weak genetic differentiation between populations are compatible with high crossing rates when it comes to orchids that do not offer rewards and deceive their pollinators, which aligns with the species under study.

Focusing on the studied subpopulations, the least differentiated are located to the west of Tenerife, comprising HCH and HBB (Guía de Isora), which are situated five kilometres apart, and HSA, which is seven kilometres away from the Guía de Isora sample sites. Orchid studies, such as those proposed by Scopece et al. (2010) [64], have shown that this low differentiation may be related to high pollinia dispersal. *Himantoglossum metlesicsianum* is a nectarless orchid; thus, its reproductive strategy relies on deceiving its pollinators. Consequently, it can be inferred that the pollinator visits the flowers of one population and, due to the absence of nectar, avoids pollinating the flowers of orchids in the same area, instead visiting other individuals that are farther away, transporting the pollinia over long distances within the pollinator’s flight range.

For *Bombus canariensis*, the primary pollinator of the species [46], a flight range of 3 to 10 km can be described, based on the related species *B. terrestris* [65]. Consequently, the pollinia of the plant could cross between different groups and nearby subpopulations. According to Pfeifer and Jentschke (2006) [23], when the distances between populations are shorter, differentiation values are lower, similar to other orchids such as *Orchis provincialis* and *Cephalanthera longifolia*. Other authors, such as Hamrick and Godt (1990) [61], agree that the unique traits of some orchid species related to deceptive pollination strategies and wind dispersal maintain a high number of populations while promoting outcrossing and gene flow between populations, aspects that may coincide with our study.

This species could exhibit two types of seed dispersal, as already detected in other orchids [66,67], characterised by a dichotomy between short and long distances, combining both. Long-distance dispersal may occur due to occasional wind phenomena, where the species seeks new opportunities to colonise new territories; meanwhile, in short-distance dispersal, new individuals are recruited around the maternal plant (HGU) [47].

In our study, the presence of significance in the values obtained for SGS (spatial genetic structure at fine scale), at distances of less than one meter, indicates the existence of relatedness among individuals in the Western Tenerife populations at distances of less than one meter (<0.55 m) for the first distance class. Although the values were significant, the coefficient of relatedness was low.

This could suggest that a portion of the seeds of *H. metlesicsianum* fall and recruit in areas close to their maternal plants, which is also related to the diminutive size of the seed and occurs in other species: *Himantoglossum hircinum*, *Orchis cyclochila*, *Cymbidium goeringii*, *Cephalanthera longibracteata*, and *Cremastra appendiculata* [50,51]. In addition, there could also be pollen dispersal primarily due to the pollinator visiting the flowering individuals of the same population.

Comparing the data obtained from the study with those proposed by Vekemans and Hardy (2004) [68], we find that, in this case, the Sp value falls within the expected ranges in the scale proposed by the authors. This is a species whose pollen dispersal occurs through animals, with the dispersal system taking place via wind and reproducing through the crossing of individuals. However, it does not align with the life form that would fit with Sp values similar to those of small trees.

A low value in the coefficient of relatedness may indicate a high gene flow between the western populations. Although it suggests that the closest individuals share a greater degree of relatedness. Other studies on orchids demonstrate that long-distance seed dispersal may occur occasionally [69] and that this low genetic differentiation may also indicate that these are initial populations of the orchid, whose long-distance dispersal events are effective, as seen in other species: *Cephalanthera rubra*, *Spiranthes romanzoffiana*, or *Orchis mascula* [70,71,72]. It is possible that among islands where the distances between populations are not relatively large (from 4 to 290 km), these events may occur at a higher frequency than expected, resulting in low genetic differentiation due to gene flow between populations and high genetic diversity values for the species in the Canary archipelago. It is important to consider the possibility that there are population nuclei close to the studied populations that are currently unknown due to difficult access; this circumstance may influence the interpretation of the obtained results.

Our genetic analyses could reveal the existence of migration between regions and effective long-distance and short-distance dispersal events. Therefore, the genetic results align with the reproductive biology of the species, as *H. metlesicsianum* combines both types of anemochorous dispersal, in addition to zoophilous dispersal among individuals of the populations.

## 4. Materials and Methods

### 4.1. Sample Collection and Genotyping

**Figure 3:**
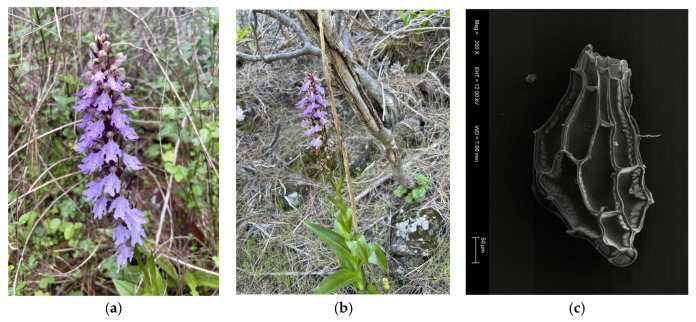
*Himantoglossum metlesicsianum*. (**a**,**b**) Flowering specimens from Güímar and Arafo in Tenerife. (**c**) Scanning electron microscope image of a seed.

*Himantoglossum metlesicsianum* is a geophyte that can grow up to one meter in height (Figure 3b). The inflorescence reaches a length of 10–20 cm; its flowers are purple with darker violet mottling, and the labellum is purple with reddish spots, measuring approximately 1.8–2.5 mm in length (Figure 3a). The fruits are capsules approximately 25 mm long, dehiscent, and characterised by four ridges where the seeds are attached. The seeds are small, with an average length of 446.34 μm and an average width of 200.27 μm (Figure 3c) [47].

**Figure 4:**
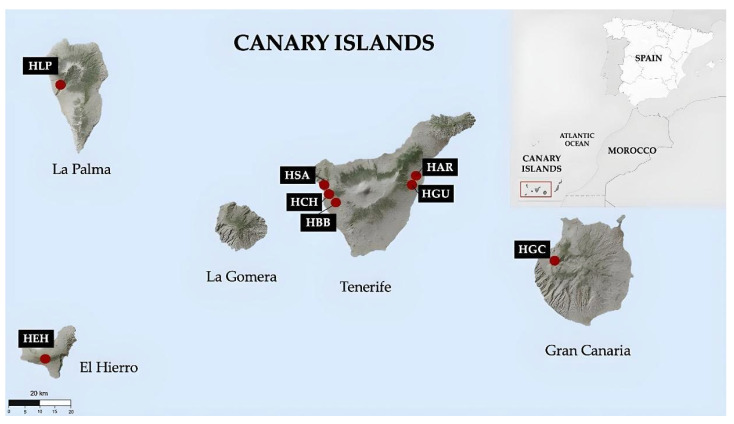
Distribution of the natural populations in the Canary Islands (red points).

In 2021, samples were collected from previously known localities: Tenerife (Arafo [HAR], Güímar [HGU], Barranco Bermejo [HBB], Chío [HCH], and Santiago del Teide [HSA]), La Palma (Tijarafe) [HLP], Gran Canaria (Tamadaba) [HGC], and El Hierro (El Pinar) [HEH] (Figure 4). On El Hierro, only one individual was recorded. After dehydration, leaf tissue from each individual was processed separately, and DNA was extracted at the individual level using a modified version of the protocol by Dellaporta et al. (1983) [73]. All specimens were georeferenced in ArcGIS (ESRI) (Table 3). Legal permission for the collections was granted by the Government of Canary Islands (Nº2020/38895).

### 4.2. Development of Microsatellites

For the species *Himantoglossum metlesicsianum*, 14 microsatellites are described to carry out the genetic characterisation of the species. During the first phase of the project, DNA samples of the species were sent to a company specialised in this field (Secugen, S.L.) for the elaboration of specific gene libraries enriched with microsatellite motifs. After obtaining the list with the primer pairs, 50 primer pairs were selected in our laboratory to test their amplification by polymerase chain reaction (PCR). The PCR products were visualised in electrophoresis gels to check for amplification and polymorphism. Afterwards, a second PCR was run with the primer pairs labelled with 6-FAM, NED, VIC, PET according to the protocol in Blacket et al. (2012) [74]. Subsequently, once the first samples had been sequenced, the most polymorphic microsatellites were filtered again. Of the 50 pairs of markers initially tested, 14 of them showed polymorphism with quality electrophoretic profiles. Once the optimal microsatellites were obtained, all the individual samples were amplified. These primers were used to genotype 95 samples from all the natural populations in the archipelago.

For final testing and genotyping, each primer pair was included in a reaction of 25 μL in total, containing approximately 1 μL of DNA, 1.62 μL of universal primer, 0.37 μL of forward primer with tail and 1.25 μL of reverse primer, 0.25 μL of BSA (bovine serum albumin) as well as PCR Master Mix to complete 25 μL (Reddy-Mix, ABgene, Surrey, UK). The amplification conditions were as follows: an initial cycle of 3 min at 95 °C, 35 cycles of 30 s at 95 °C, 30 s at 59 °C, 1 min at 72 °C, and a final cycle of 5 min at 72 °C. The second condition was an initial cycle of 3 min at 95 °C, 35 cycles of 30 s at 95 °C, 30 s at 58 °C, 1 min at 72 °C and a final cycle of 5 min at 72 °C. Finally, an initial cycle of 3 min at 95 °C, 35 cycles of 30 s at 95 °C, 30 s at 57 °C, 1 min at 72 °C, and a final cycle of 5 min at 72 °C.

PCR products from both simple reactions were analysed using a Genetic Analyzer, and fragment sizes were calibrated using the GS500(-250)LIZ size standard (Applied Biosystems, Inc., Foster City, CA, USA.). Capillary sequencing data were analysed using the Microsatellite Analysis (MSA) online tool provided by Thermo Fisher Scientific (Waltham, MA, USA). We determined allele peak profiles for each locus and subsequently assigned genotypes to all individuals. The sequences were deposited in GenBank (Table S1 in Supplementary Material).

### 4.3. Genetic Analysis

The deviation from Hardy–Weinberg equilibrium and linkage disequilibrium were calculated using Genepop 4.2 software [75]. For this, exact probability tests according to the Markov chain method [76] were used. The fixation index (*F_IS_*) was calculated to measure the magnitude of the deviation from Hardy–Weinberg equilibrium, a value obtained by the method described by Weir and Cockerham (1984) [77]. The estimation of null alleles was carried out with MICRO-CHECKER 2.2.3 software implementing the Oosterhout algorithm [78].

Basic genetic diversity, such as allele frequencies, mean number of alleles per locus (NA), allele richness (A), observed heterozygosity (H_o_), expected heterozygosity (H_e_) for each locus and population were estimated using GenAlEx software version 6.5 [79]. Measures of allelic richness (Ar) and the richness of exclusive alleles were calculated using HP-RARE 1.0 [80]. This software applies rarefaction to correct for differences in sample size, standardising to the smallest sample size, which in this case was 10 genes.

Estimates of selfing (Sr) were calculated for each population and implemented in SPAGeDi 1.5 [68] with the method described in David et al. (2007) [81].

The hierarchical structure was inferred through analysis of molecular variance (AMOVA) [82] with Arlequin software version 3.0 [83]. Significance values were obtained on 100 combinations. In this case, two analyses have been carried out: (1) all the populations of each island (Tenerife, La Palma and Gran Canaria) without the locality of El Hierro (2) all the populations of Tenerife (West group: HBB, HCH, HSA and East group: HAR and HGU). In the first AMOVA analysis, four groups were established, taking into account the results obtained in the STRUCTURE 2.3.4 software.

The degree of genetic differentiation between populations was calculated with GenAlEx software version 6.5 [79], estimating the *F_ST_* (genetic differentiation coefficient). The study of the population genetic structure of the species was estimated using STRUCTURE software [84], and the genotypes were analysed using the Bayesian clustering model. The procedure is performed with 10 independent replicates for each K value (1 to 10). The analysis consisted of an initial burn-in period of 105 replicates/replicates and a subsequent run with 106 Markov chain interactions (MCMC). The optimal number of clusters was estimated using the ΔK method visualised in Structure Harvester [85,86]. The results obtained for the optimal K were processed from CLUMPP 1.1.2 [87]

In addition, a PCoA was also performed, making use of the standardised covariance method of genotypic distances between individuals and populations, which was implemented using GenAlEx 6.5 [79]. Another analysis performed to corroborate the genetic structure detected in STRUCTURE is the discriminant analysis of principal components (DAPC). This analysis looks for a reduced space in which observations are best discriminated into predefined groups [88]. The analyses have been implemented with R software, with the Adegenet statistical package [89].

To establish the genetic relationships among populations and individuals, neighbour-joining (NJ) dendrograms were implemented. The genetic distance between populations was calculated using Nei’s genetic distance matrix between the different locations, which was estimated using Populations 1.2 software [90] with a bootstrap of 100 for each locus and the analysis was based on Nei’s distance. As a result, a tree was obtained, which was visualised using the FigTree software [91].

In order to study fine-scale genetic structure (SGS), kinship coefficients (F_ij_) were inferred in SPAGeDi. The degree of significance of spatial genetic structure (SGS) was assessed by testing the slope of the distance regression (blin) with 10,000 permutations. The Sp statistic (a measure of the strength of SGS) was obtained by the formula Sp = –b/(1-F1), where F1 is the kinship coefficient in the first distance class following Vekemans and Hardy (2004) [68]. In this study, F1 corresponds to the first kinship coefficient within groups. Genetic coancestry indices were averaged over a set of ten distance classes, automatically defined to contain the same number of pairwise comparisons within each distance interval and regressed on the natural logarithm of the spatial distance between individuals (*lnd_ij_*) to calculate the regression slope (*blog*). To obtain 95% confidence intervals around the null hypothesis of random genetic structure, 10.000 random permutations of individual locations were tested. For this analysis, only the UTM coordinates of each of the individuals in the population nuclei located in the west of Tenerife were taken into account: HSA, HCH and HBB. Based on the results obtained in the study and the proximity of the individuals, 45 individuals were selected from the aforementioned subpopulations.

## 5. Conclusions

This study provides the genetic diversity and population genetic structure of *Himantoglossum metlesicsianum*, an endangered orchid endemic to the Canary Islands. Despite its small and fragmented populations, the species shows moderate to high genetic diversity, especially in Western Tenerife. Genetic analyses revealed low differentiation among populations, suggesting that long-distance seed and pollen dispersal play a key role in maintaining gene flow across the islands.

The highest genetic differentiation was found between La Palma and Gran Canaria, likely due to geographic distance. In contrast, populations in Western Tenerife are genetically similar, potentially due to the absence of physical barriers and frequent reproductive exchange. Spatial autocorrelation revealed a fine-scale genetic structure with low kinship, indicating both restricted local seed dispersal and effective long-distance dispersal mechanisms.

The critically endangered El Hierro population, consisting of a single individual, is genetically linked to Western Tenerife, suggesting colonisation via long-distance dispersal. These results highlight the importance of conserving natural dispersal pathways to ensure population resilience.

Further studies incorporating broader sampling and genomic tools are needed to refine conservation strategies and safeguard the long-term viability of *H. metlesicsianum* in its native habitats.

### Implications for Conservation

Understanding the genetic diversity and structure of populations is a fundamental tool for designing conservation plans for species that are threatened [92]. These studies provide insights into the current status of the species under investigation. The species is significantly impacted by illegal collection, habitat destruction, primarily due to the construction of new roads, and by the resumption of agricultural activities (vineyard cultivation) in previously abandoned plots.

*Himantoglossum metlesicsianum* appears in national and European catalogues with the category “Endangered”. This study reaffirms this category, given that over the years, the number of individuals detected has decreased or remained constant. Some populations are highly endangered, such as the locality of El Hierro, where only one individual has been counted in the last three years.

The importance of creating a recovery plan for the species and obtaining updated censuses for the Canary Islands is highlighted. To reinforce the population of the species, it is essential to consider the nearest origin of the individuals. For the western region of Tenerife, this study demonstrates a genetic relationship, which could allow for population reinforcement in those locations.

It is essential to ensure the protection of the areas currently inhabited by the species, with the aim of safeguarding its presence, as well as its pollinators and the dispersal of seeds to new areas, thereby promoting the gene flow of the taxon. Furthermore, it will be necessary to develop an optimised protocol for ex situ conservation through in vitro cultivation [47] and to analyse the soil where the species resides to understand the existing relationship between its roots and the presence of mycorrhizae that ensure its successful growth and development. Environmental awareness campaigns are essential for disseminating information about the plant and preventing the illegal collection and cutting of its flowering stems, particularly in areas where the species coexists with human activities, mainly tourism (such as trails) and agriculture (such as vineyards).

Delimiting the territory by fencing off individual plots of land where the species occurs can help address the issues related to collecting in those more well-known populations. Environmental awareness campaigns are also necessary to disseminate information about the plant and to avoid illegal collection and cutting of its flowering stems.

## Figures and Tables

**Table 1:** Genetic diversity indices by island and population of the species *Himantoglossum metlesicsianum*. N: Number of samples. N_a_: Average number of alleles per locus. A_r_: Allelic richness. Rar: Allelic richness with rarefaction (10 genes). PA: Number of private alleles. NTA: Total number of alleles. *H_o_*: Observed heterozygosity. *H_e_*: Expected heterozygosity. *F_IS_*: Fixation index. S_r_: Selfing rates. P (%): Percentage of locus polymorphism by population.

Population	N	N_a_	A_r_	Rar	PA	NTA	*H_o_*	*H_e_*	*F_IS_*	S_r_	P (%)
HAR	14	4.929	3.52	0.19	2	69	0.509	0.612	0.000 *	0.188	100%
HBB	15	5.643	4.13	0.41	4	79	0.686	0.695	0.012 ^ns^	-	93%
HCH	15	5.572	3.99	0.26	4	78	0.644	0.677	0.0069 ^ns^	0.040	100%
HGU	15	4.286	3.4	0.11	1	60	0.657	0.617	0.0096 ^ns^	0.136	100%
HSA	15	5.571	4.03	0.46	6	78	0.667	0.694	0.0103 ^ns^	0.200	100%
HGC	13	5.50	4.01	0.43	4	77	0.533	0.619	0.000 **	0.087	100%
HLP	7	3.714	3.37	0.14	1	52	0.503	0.568	0.0004 **	-	93%
Total	94		-	-	39	128					

Statistical significance: * indicates *p* < 0.05; ** indicates *p* < 0.01; (^ns^) denotes non-significant differences.

**Table 2:** AMOVA analysis for the species *Himantoglossum metlesicsianum*.

Source of Variation	Degrees of Freedom	Sum of Squares	Components of Variance	Percentage of Variation	F-Statistics
TENERIFE, LA PALMA AND GRAN CANARIA					
Between islands	2	45.619	0.341	7.81	*F_CT_* = 0.067 ***
Between populations within islands	4	58.491	0.370	8.46	*F_SC_* = 0.116 ***
Within populations	181	663.251	3.664	83.73	*F_ST_* = 0.175 ***
Total	187	767.362	4.376		
TENERIFE [HAR, HBB, HCH, HGU, HSA]					
Between groups (West vs. East Tenerife)	1	22.487	0.127	2.76	*F_CT_* = 0.097 ***
Between populations within groups	3	40.858	0.319	6.94	*F_SC_* = 0.071 ***
Within populations	143	593.743	4.152	90.30	*F_ST_* = 0.02 ***
Total	147	657.088	4.598		
Statistical significance: *** indicates *p* < 0.001					

**Table 3:** Localisation of *Himantoglossum metlesicsianum* populations. Approximate UTM coordinates are shown. Total number of individuals per population detected in the 2021 census and number of individuals analysed during this study. The population codes represent the acronyms of their respective populations.

Population	Population Code	UTM Coordinates	Total Number Individuals	Number of Individuals Analysed
Arafo	HAR	357000 3134500	21	14
Barranco Bermejo	HBB	329500 3123000	20	15
Chío	HCH	325500 3126500	41	15
Santiago del Teide	HSA	323000 3132000	84	15
Güímar	HGU	357000 3134000	116	15
La Palma	HLP	213000 3181500	7	7
Gran Canaria	HGC	429500 3104000	14	13
El Hierro	HEH	199500 3069500	1	1

## Data Availability

The data presented in this study are available from the corresponding author upon reasonable request.

## Supplementary Materials

The following supporting information can be downloaded at: https://www.mdpi.com/article/10.3390/plants14121862/s1, Table S1: Polymorphic microsatellites developed and obtained for *Himantoglossum metlesicsianum*. GENBANK ID: Identifier code of each locus in the Genbank database. Primer set in the reaction: forward primer (F), reverse primer (R). Table S2: Values of the genetic differentiation coefficient (F_ST_) between the analysed populations of *Himantoglossum metlesicsianum*. All F_ST_ values were significant (*p* < 0.05)., Figure S1: Principal Component Analysis (PCoA) at the population level obtained from the GenAlEx 6.5 software. Table S3: Evanno table obtained from Structure Harvester. Figure S2: K values calculated from ln(K) and K using Structure Harvester. Figure S3: BIC value versus the number of groups (clusters) in the Structure Harvester analyses.
