# Supplementary figures and images for: Endangered with High Dispersal Abilities: Conservation Genetics of Himantoglossum metlesicsianum (Teschner) P. Delforge (Orchidaceae) in the Canary Islands
Source: Plants (Basel). 2025 Jun 17;14(12):1862. doi: 10.3390/plants14121862 (PMC12197004; doi:10.3390/plants14121862)

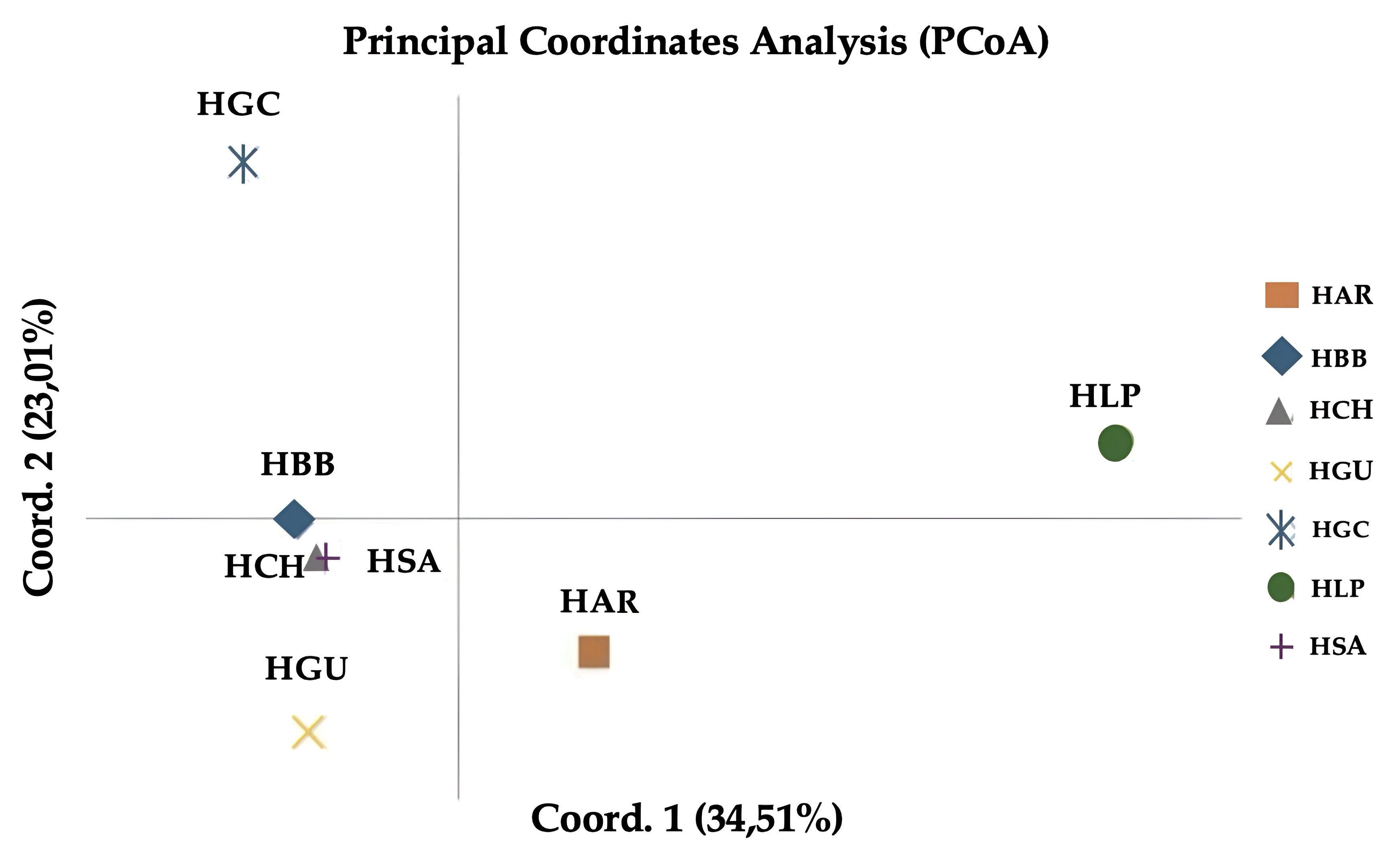

Supplement: Supplementary file 1 [file plants-14-01862-s001.zip › Figure S1. Principal Component Analysis (PCoA)jpg.jpg]

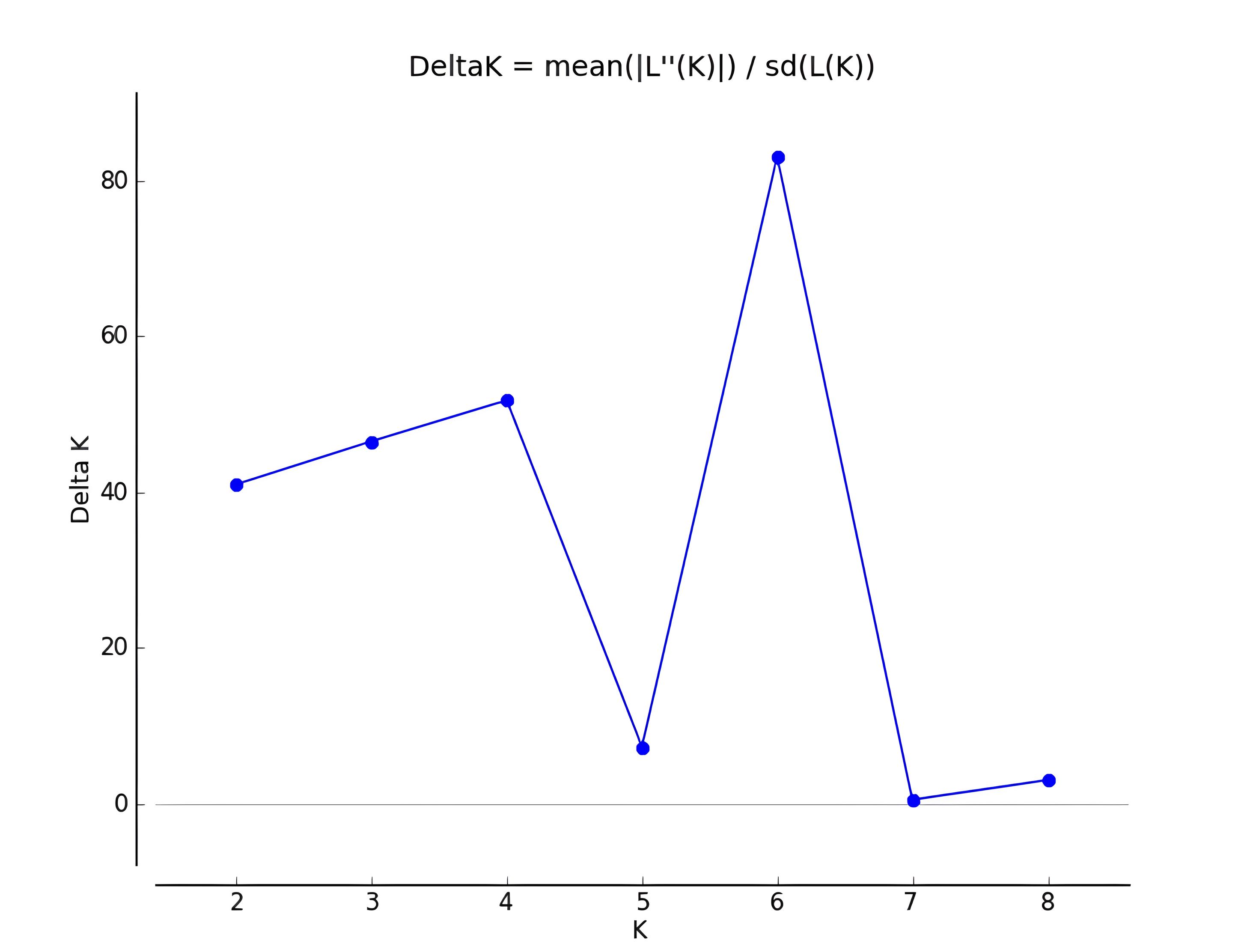

Supplement: Supplementary file 1 [file plants-14-01862-s001.zip › Figure S2. K values calculated from ln(K) and K using Structure Harvester.jpg]

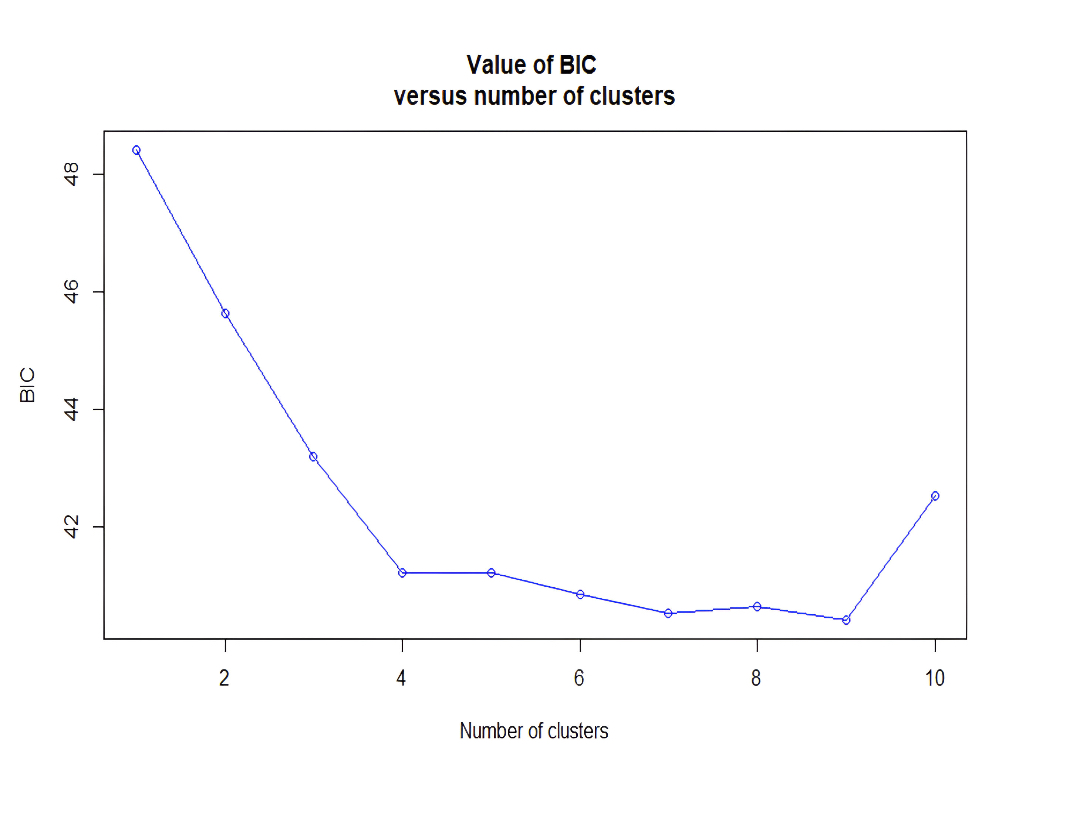

Supplement: Supplementary file 1 [file plants-14-01862-s001.zip › Figure S3. BIC value versus the number of groups (clusters) in the Structure Harvester analyses.jpg]

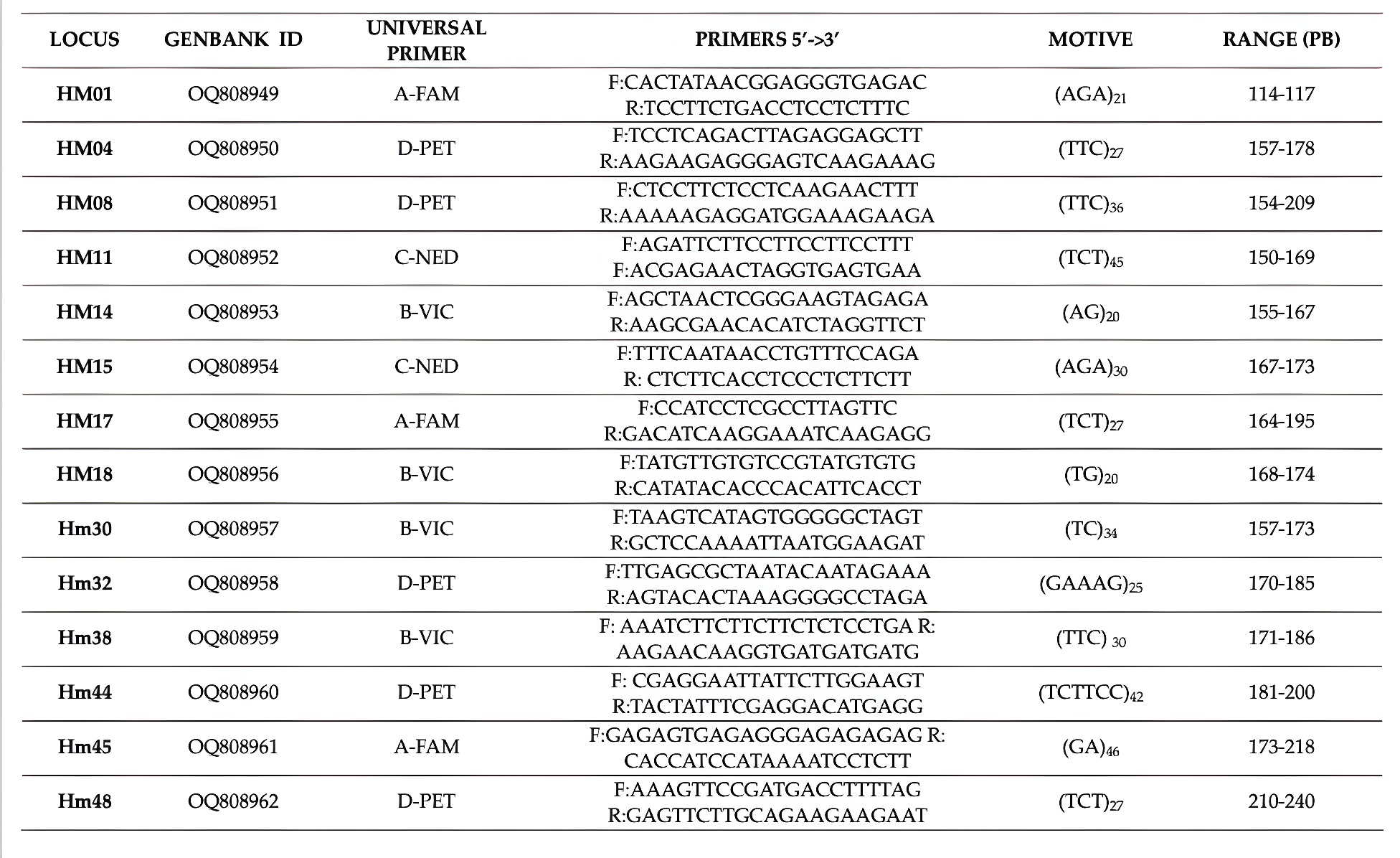

Supplement: Supplementary file 1 [file plants-14-01862-s001.zip › Table S1. Polymorphic microsatellites .jpeg]

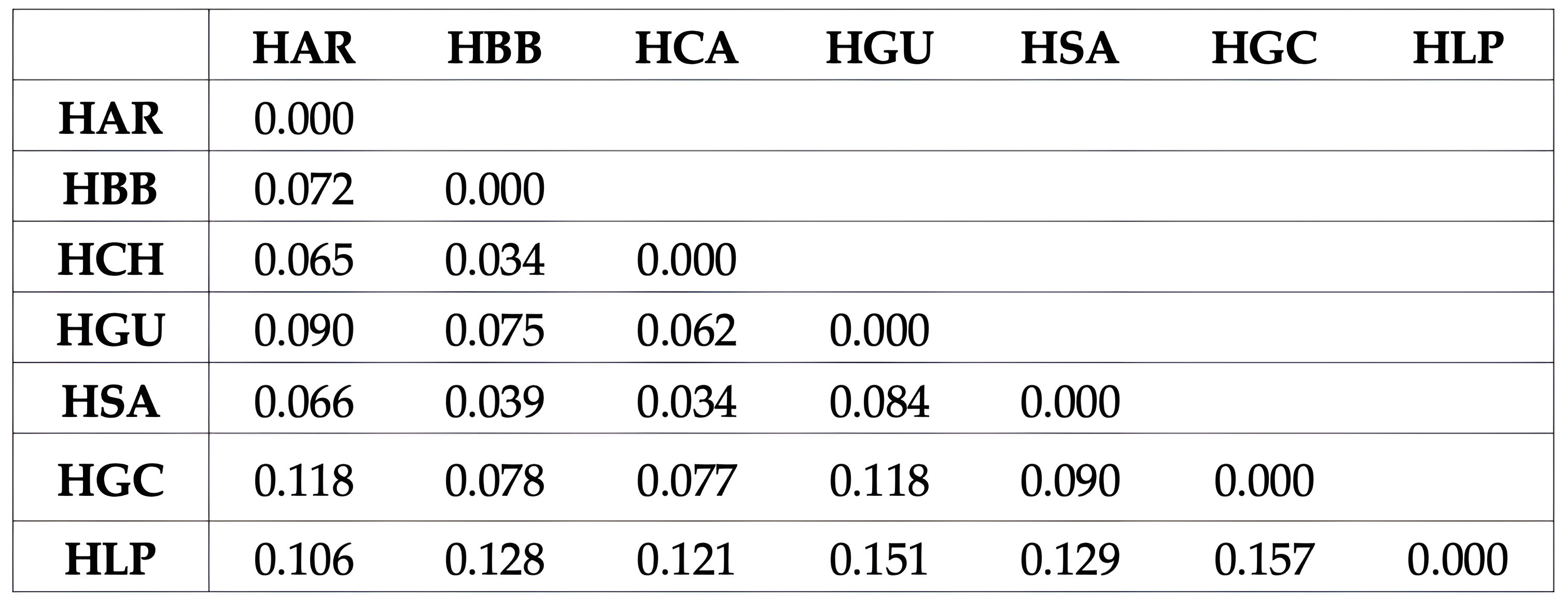

Supplement: Supplementary file 1 [file plants-14-01862-s001.zip › Table S2. Values of the genetic differentiation.jpg]

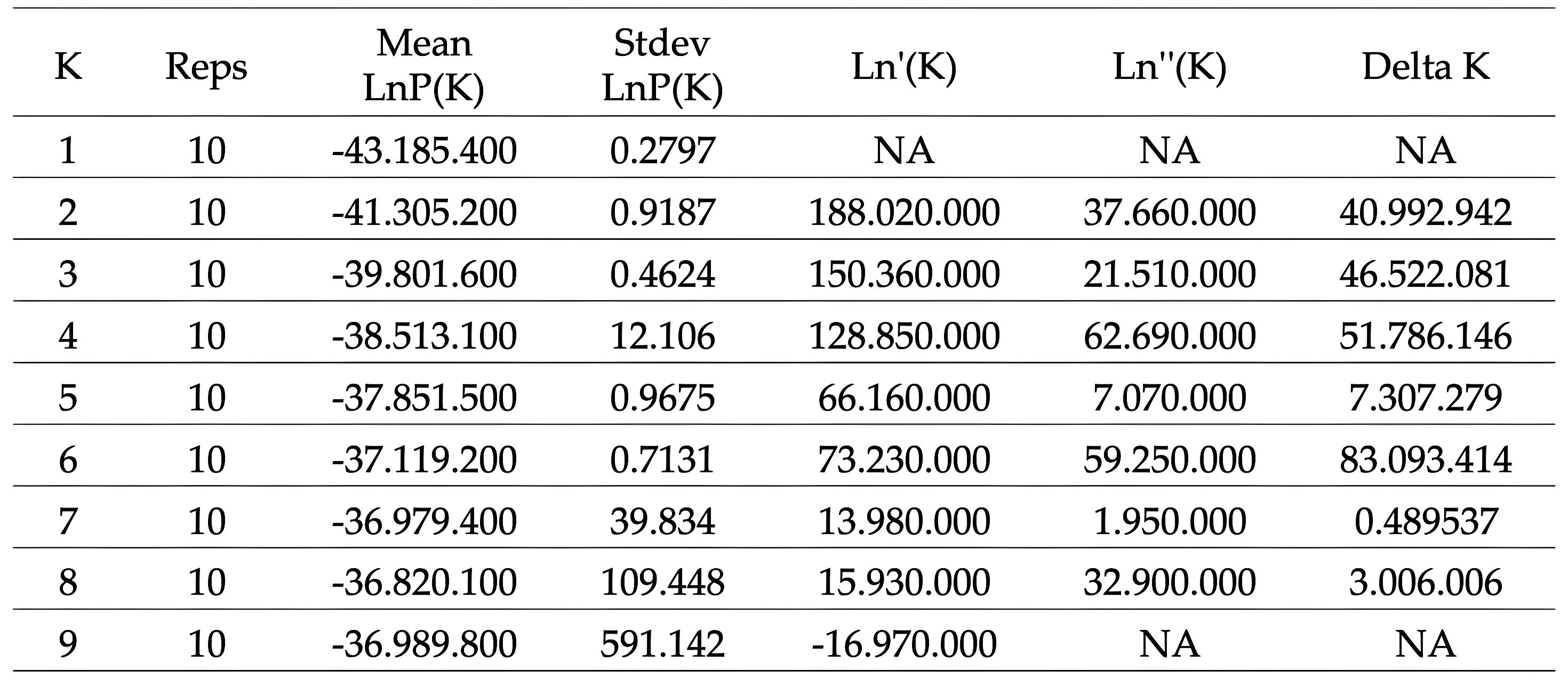

Supplement: Supplementary file 1 [file plants-14-01862-s001.zip › Table S3. Evanno table obtained from Structure Harvester.jpeg]
